# Therapeutic Potentials of Poly (ADP‐Ribose) Polymerase 1 (PARP1) Inhibition in Multiple Sclerosis and Animal Models: Concept Revisiting

**DOI:** 10.1002/advs.202102853

**Published:** 2021-12-21

**Authors:** Yan Wang, David Pleasure, Wenbin Deng, Fuzheng Guo

**Affiliations:** ^1^ Department of Neurology School of Medicine University of California Davis CA 95817 USA; ^2^ Institute for Pediatric Regenerative Medicine UC Davis School of Medicine/Shriners Hospitals for Children Sacramento CA USA; ^3^ School of Pharmaceutical Sciences (Shenzhen) Sun Yat‐sen University Guangzhou 510006 China

**Keywords:** demyelination, inflammatory demyelinating disease, multiple sclerosis, neuroinflammation, neuronal/axonal degeneration, PARP1, poly‐ADP ribosylation

## Abstract

Poly (ADP‐ribose) polymerase 1 (PARP1) plays a fundamental role in DNA repair and gene expression. Excessive PARP1 hyperactivation, however, has been associated with cell death. PARP1 and/or its activity are dysregulated in the immune and central nervous system of multiple sclerosis (MS) patients and animal models. Pharmacological PARP1 inhibition is shown to be protective against immune activation and disease severity in MS animal models while genetic PARP1 deficiency studies reported discrepant results. The inconsistency suggests that the function of PARP1 and PARP1‐mediated PARylation may be complex and context‐dependent. The article reviews PARP1 functions, discusses experimental findings and possible interpretations of PARP1 in inflammation, neuronal/axonal degeneration, and oligodendrogliopathy, three major pathological components cooperatively determining MS disease course and neurological progression, and points out future research directions. Cell type specific PARP1 manipulations are necessary for revisiting the role of PARP1 in the three pathological components prior to moving PARP1 inhibition into clinical trials for MS therapy.

## Graphical abstract

PARP1 inhibitors have been approved for treating cancer patients. The therapeutic potential of PARP1 inhibition in multiple sclerosis, an inflammatory demyelinating disorder of the central nervous system remains elusive. This review highlights the current state and controversies, presents possible interpretations and hypotheses, and points out future research directions in the field of PARP1 regulation of multiple sclerosis pathogenesis.

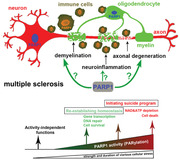

## 1 Introduction

Multiple sclerosis (MS) is a chronic inflammatory demyelinating disease of the central nervous system (CNS) affecting ≈2.5 million people around the world. The FDA‐approved disease‐modifying therapies slow MS disease relapses through suppressing immune activation,^[^ ^1^ ^]^ but they are ineffective in preventing neurological progression. While chronic neuronal/axonal degeneration is the proximate cause of neurological progression, mounting evidence from animal studies suggests that myelin repair or remyelination could prevent axonal loss and promote functional recovery.^[^ ^2^ ^]^ Therefore, the combined immune‐regulatory and myelin repair‐promoting therapies represent the promising therapeutic options for MS. Discovering molecular targets that exert multi‐modal actions on immune regulation, oligodendroglial demyelination/remyelination, and neuronal/axonal degeneration will provide novel insights into MS therapies. PARPs (a.k.a. ADP ribosyl‐transferases diphtheria toxin‐like, ARTDs) is one example which has been reported to regulate inflammation, myelin damage and repair, and neuronal and axonal damage in different contexts. Importantly, PARP inhibitors have been approved by FDA for treating homologous recombination‐deficient cancer patients^[^ ^3^ ^]^ indicating a safety profile.

PARP1 (a.k.a. ARTD1) is the founding member of PARP family and the most abundant nuclear protein after histones (≈1 million molecules per cell, equivalent to 1 molecule per 22 nucleosomes) and plays a role in a variety of fundamental cellular processes, such as DNA repair and genomic stability maintenance,^[^ ^4^ ^]^ chromatin remodeling,^[^ ^5^ ^]^ gene expression,^[^ ^6^ ^]^ cell differentiation,^[^ ^7^ ^]^ and cell survival and death.^[^ ^8^ ^]^ Our knowledge of PARP1's functions is still expanding^[^ ^9^ ^]^ and we recommend readers refer to excellent review articles covering different functional aspects. In the CNS, PARP1 has been proposed as a potential therapeutic target in animal models of traumatic^[^ ^10^ ^]^ and ischemic^[^ ^11^ ^]^ brain injuries and neurodegenerative disorders as well.^[^ ^12^ ^]^

Recently, PARP1 mRNA was increased in T and B lymphocytes.^[^ ^13^ ^]^ PARP1 enzymatic activity was also upregulated in myeloid cells of the peripheral blood of MS patients.^[^ ^14^ ^]^ Furthermore, PARP1 activation was detected in oligodendrocytes (OLs), and to a lesser extent, in astrocytes and microglia/macrophages in the active areas of brain lesions of MS patients.^[^ ^15^ ^]^ Using a non‐human primate MS model of experimental autoimmune encephalomyelitis (EAE), it was reported that PARP1 activation was detected primarily in astrocytes surrounding demyelination plaques and in scattered nearby microglia, OLs, and neurons.^[^ ^16^ ^]^ These clinical and preclinical findings suggest that PARP1 may be a therapeutic target for intervening neuroinflammation (see review^[^ ^17^ ^]^) and possibly oligodendrogliopathy and neuropathy/axonopathy in MS patients and animal models. It is noteworthy that most previous studies employed pharmacological inhibitors to interrogate the role of PARP1 in MS pathogenesis.^[^ ^17^ ^]^ However, the broad expression of PARP1 in different immune and neural cell types makes it imperative to use cell‐specific approaches and probe the context‐dependent role of PARP1 in MS pathologies. In this article, we highlight the basic function and potential mechanisms of PARP1 in regulating immune response and neuroinflammation, neuronal damage and axonal degeneration, and oligodendroglial biology and pathology. We discuss the effects of PARP1 chemical inhibition and genetic deficiency on pathological presentations of MS‐modeled animals, point out strengths and weaknesses of available experimental data in the field, and present possible alternative interpretations underlying apparently discrepant findings.

### 1.1 Animal Models of MS

Though infiltrated inflammatory cells (T lymphocytes and macrophages) are present in the CNS of all MS patients regardless of disease course and duration, four fundamentally different patterns of demyelination lesions are observed, indicative of different pathogenesis.^[^ ^18^ ^]^ Pattern I and II plaques resemble autoimmune encephalomyelitis (pattern I – T cell‐mediated cellular immunity and pattern II – antibody‐mediated humoral autoimmunity) while pattern III and IV plaques display similarity to primary oligodendrogliopathy (pattern III – OL apoptosis and pattern IV – OL dystrophy). Therefore, the most popular animal models in MS research could be divided into two fundamental categories: EAE, which mimics T‐cell‐mediated autoimmunity (Pattern I MS lesions), and toxin‐induced demyelination, which mimics primary oligodendrogliopathy (Pattern III and IV lesions).

1) EAE models involve actively immunizing animals (most commonly mice, rats, and non‐human primates) with myelin proteins or peptides in conjunction with strong immune‐stimulating adjuvants, in most cases complete Freund's adjuvant to induce myelin‐specific autoreactive T cell responses. The EAE model is the most common in vivo system to study the mechanisms and regulations of immunological aspects and brain inflammation of MS patients. In the murine EAE model, immunization of different genetic background mice with different myelin protein peptides generates EAE displaying different MS‐like disease courses. For example, C57BL/6 mice can be immunized with myelin OL glycoprotein peptide 35‐55 (MOG‐peptide_35‐55_) to elicit an acute neuroinflammatory infiltration followed by progressive neurodegeneration,^[^ ^19^ ^]^ mimicking chronic progressive MS patients whereas SJL mice that are immunized with proteolipid protein peptide139‐151 (PLP‐peptide_139‐151_) elicit a course resembling relapsing‐remitting MS, the most common form of MS. It has been reported that immunizing nonobese diabetic (NOD) mice with MOG‐peptide_35‐55_ generates an MS‐like disorder mimicking secondary progressive MS disease course characterized by multiple immune attacks and remissions followed by a progressively worsening phase.^[^ ^20^ ^]^ However, recent experimental data challenged this and reported that MOG‐peptide_35‐55_‐EAE of NOD mice is not a progressive model.^[^ ^21^ ^]^ MOG‐peptide_35‐55_ immunization to 129S genetic background mice produces mild to moderate EAE compared to C57BL/6 mice.^[^ ^22^ ^]^ It should be noted that the canonical EAE model primarily generates a major histocompatibility complex (MHC) Class II‐restricted, CD4^+^ T cell response with minimal CD8^+^ T cell or B cell involvement, in sharp contrast to MS patients where CD8^+^ T cells and B cells play an essential role in pathogenesis. A variant of EAE model is passive transfer of autoreactive T cells (CD4^+^ or CD8^+^) alone or together with pathogenic autoantibodies to naïve recipient mice, eliciting autoimmune demyelination lesions in recipient mice. Another useful animal model for studying MS pathology is chronic inflammatory demyelination disease induced by the JHM strain of murine hepatitis virus and characterized by chronic inflammation and formation of primary demyelination plaques with variable axonal damage and loss.^[^ ^23^ ^]^
2) The most popular toxin‐induced demyelination models include diffuse demyelination model elicited by cuprizone diet and focal demyelination elicited by local injections of lysophosphatidylcholine (LPC, lysolecithin) or ethidium bromide (EB). In the cuprizone model, adult rodents (mice or rats) are fed 0.2–0.3% copper‐chelating cuprizone (w/w) in chow for 4–6 weeks. The chow selectively damages mature OLs, likely through disrupting mitochondrial function, and ultimately leads to demyelination primarily in the corpus callosum. There is robust activation of resident microglia yet without substantial inflammatory infiltrations of peripheral macrophages,^[^ ^24^ ^]^ T lymphocytes, or B lymphocytes^[^ ^25^ ^]^ in the demyelinating area of cuprizone‐fed animals. A recent study reported the presence of T cells in cuprizone‐induced demyelination area despite the number being low.^[^ ^26^ ^]^ Interestingly, nonhuman primates are resistant to cuprizone‐induced brain demyelination,^[^ ^27^ ^]^ suggesting an intrinsic species difference. The focal demyelination model, which is induced local CNS injection of LPC, a non‐specific lipid and membrane‐disrupting molecule,^[^ ^28^ ^]^ elicits the death and damage of all types of cells including OLs and myelin in the injection site. Different from cuprizone model, there is a robust yet transient infiltration of peripheral myeloid^[^ ^29^ ^]^ and lymphoid cells^[^ ^30^ ^]^ into the LPC‐induced demyelination area. Unlike inflammatory EAE models, successful OL regeneration and remyelination occur in toxin‐induced diffuse and focal demyelination models, thus making them useful in interrogating cellular and molecular pathways underlying CNS myelin repair.

## 2 PARP1‐Mediated PARylation: Functions and Regulations

**Figure 1:**
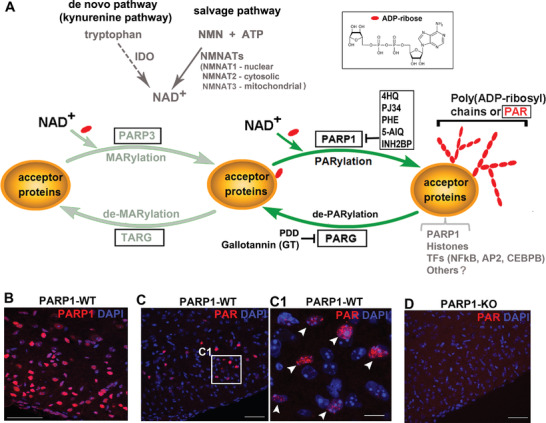
ADP‐Ribosylation and representative catalytic enzymes. A) Protein ADP‐ribosylation consists of mono‐ADP‐ribosylation (MARylation) and poly‐ADP‐ribosylation (PARylation) which are catalyzed by mono‐ADP‐ribosyl transferases (such as PARP3) and poly‐ADP‐ribosyl transferases (such as PARP1), respectively, by using NAD^+^ as the donor of ADP‐ribose unit. NAD^+^ is replenished and synthesized primarily through the salvage pathway catalyzed by the rate‐limiting enzyme NMNATs and, to a lesser extent, through the de novo kynurenine pathway catalyzed by the rate‐limiting enzyme IDO. B–D) immunohistochemical staining of PARP1 and PAR in the spinal cord of PARP1 wild type(WT) and PARP1 knockout(KO) mice of postnatal 10 days. Boxed area in (C) is shown at higher magnification images in (C1). Note that PARP1 deficiency abolished the nuclear PAR signal, suggesting that PARP1 is the predominant PARP responsible for PARylation in the CNS. Scale bars: 100 µm, (B,C,D); 10 µm, (C1). **Abbreviations**: NAD^+^, nicotinamide adenine dinucleotide; MNN, nicotinamide mononucleotide; ATP, adenosine triphosphate; PARG: poly ADP‐ribose glycohydrolase; TARG, terminal ADP‐ribose glycohydrolase; IDO, indoleamine 2,3‐dioxygenase; NMNATs, nicotinamide mononucleotide adenylyltransferase. PARP1 inhibitors: 4HQ, 4‐hydroxy‐quinazoline; PJ34, *N*‐(6‐oxo‐5, 6‐dihydrophenanthridin‐2‐yl)‐(*N*,‐dimethylamino) acetamide hydrochloride; PHE, 5(5H)‐phenanthridinone; 3AB, 3‐aminobenzamide; 5‐AIQ, 5‐aminoisoquinoline. PDD, PDD 00017273, a potent and selective inhibitor for PARG.

PARPs consist of 17 members in mammals and share a conserved kinase domain of ADP‐ribosyl‐transferase activity. Despite being referred to as PARPs, only four members, PARP1, PARP2, PARP5a (aka Tankyrase 1), and PARP5b (aka Tankyrase 2) catalyze the covalent attachment of poly(ADP‐ribose) units (PAR) to acceptor proteins, a process called PARylation, whereas the majority (PARP3, PARP4, PARP6, PARP7‐12, and PARP14‐16) catalyze mono(ADP‐ribose) addition to target proteins (MARylation) (**Figure** **1**A). PARP family members show differential subcellular localizations. For example, PARP1 is strictly localized in the nucleus (**Figure** 1B); PARP2 and PARP3 are predominantly localized in the nucleus; PARP5a/5b and PARP14 are predominantly in the cytoplasm;^[^ ^31^ ^]^ and PARP16 is localized in the endoplasmic reticulum (ER).^[^ ^32^ ^]^ These differential subcellular localizations suggest the different primary functions of each PARP member.

Protein PARylation is a type of posttranslational modification that plays a crucial role in regulating diverse biological processes in response to physiological and pathological stimuli. PARP1 accounts for ≈90% of PARylation in the cells under genotoxic conditions.^[^ ^33^ ^]^ The catalytic activity of PARP1 results in the deposits of linear or branched PAR chains (with up to 200 units) onto its nuclear acceptor proteins (also referred to as target proteins). Detecting the PAR chains using specific antibodies provides a reliable surrogate for PARP1 enzymatic activity. It is well‐established that PARP1 is activated by DNA breaks and plays an essential role in DNA repair and genomic integrity maintenance.^[^ ^5^ ^]^ Using PAR‐specific antibodies, we found that PARP1 was activated in the CNS during postnatal normal development (Figure 1C–C1) where genotoxic stress is minimal. Nuclear PAR^+^ signal was completely abolished in PARP1‐KO mice (Figure 1D). These observations indicate that PARP1 is the major PARP member that contributes all cellular PARylation in the normal developing CNS.

The biological functions of PARP1 are primarily through its PARylation activity although activity‐independent roles are not uncommon, for example, functioning as a chromatin‐binding protein.^[^ ^34^ ^]^ Because of the structural similarity of PAR to DNA/RNA and the bulky negative charges carried by PAR chains, PARP1‐mediated PARylation modulates the functions of target proteins by preventing binding of PARylated proteins to their DNA/RNA partners via electrostatic repulsion, affecting important protein‐protein interactions via topological changes, modulating the biochemical properties of PARylated proteins themselves, employing the PAR‐chains as scaffolds for recruiting other nuclear proteins, and other yet unidentified mechanisms.^[^ ^35^ ^]^ Among the known acceptor proteins are PARP1 itself,^[^ ^36^ ^]^ histones,^[^ ^37^ ^]^ and transcription factors for instance nuclear factor *κ*B (NF‐*κ*B),^[^ ^38^ ^]^ activating protein 2 (AP2),^[^ ^39^ ^]^ CCAAT/enhancer‐binding protein beta (CEBP/*β*),^[^ ^40^ ^]^ and those participating in DNA damage response and repair. PARP1 itself is the prime PAR acceptor protein in cells and PARP1 auto‐PARylation provides a negative feedback limiting its PARylation activity. In addition, the steady‐state levels of the cellular nicotinamide adenine dinucleotide (NAD^+^) molecules modulate PARP1 activity (**Table** **1**). Cellular NAD^+^ level is replenished and maintained by two biosynthesis pathways: the salvage pathways in which compartmentalized nicotinamide mononucleotide adenylyltransferases (NMNATs) are key enzymes^[^ ^41^ ^]^ and the de novo pathway in which indoleamine 2,3‐dioxygenase (IDO) catabolizes tryptophan into NAD^+^ through multiple steps (IDO‐kynurenine pathway) (**Figure** 1A). Mammalian cells predominantly rely on the salvage pathway for NAD^+^ biosynthesis.^[^ ^41^ ^]^ In this regard, it is not surprising that another family of NAD^+^‐consuming enzymes Sirtuins antagonize PARP1 activity^[^ ^42^ ^]^ (Table 1). It has been shown that the histone variant macroH2A1.1 acts as an endogenous inhibitor for PARP1 activity^[^ ^43^ ^]^ and potentially limits nuclear NAD^+^ consumption.^[^ ^44^ ^]^ Together, multiple mechanisms exist in cells to control PARP1 hyperactivation.

**Table 1:** Examples of endogenous triggers of PARP1 activity and possible implications in MS pathology

Upstream modulators	PARP1 modulation	Possible implications of PARP1 modulators in MS pathology
Reactive oxygen species (ROS)	Activation	Oxidative and nitrosative stress induces DNA damages and is involved in MS pathogenesis^[^ ^52^ ^]^
Reactive nitrogen species (RNS)	Activation	Substantial DNA damage has been observed in the MS and EAE mice^[^ ^53^ ^]^
Extracellular signal‐regulated kinases (ERK1/2). (Activated PARP1 further enhances ERK1/2 activity, providing a feed‐forward loop)	Activation^[^ ^54^, ^55^ ^]^	ERK1/2 activation promotes OL differentiation and myelination,^[^ ^56^ ^]^ enhances microglial and astroglial activation, presumably augmenting neuroinflammation,^[^ ^57^ ^]^ promotes neuronal survival or death in a context‐dependent manner^[^ ^55^, ^58^ ^]^
Neurotransmitter serotonin (5‐HT)	Activation^[^ ^59^ ^]^	Serotonin levels are diminished in MS patients/5‐HT may exert immunosuppressive and neuroprotective effects^[^ ^60^ ^]^
Tumor necrotic factor alpha (TNF*α*)	Activation^[^ ^61^ ^]^	Tumour necrosis factor alpha (TNFα), upregulated in MS patients and animal models, is required for oligodendroglial remyelination,^[^ ^62^ ^]^ regulates neuroinflammation,^[^ ^63^ ^]^ and exerts neuroprotection^[^ ^64^ ^]^
Neuronal activity, molecularly mediated by phospholipase C (PLC) and calmodulin‐dependent kinase II (CaMKII)	Activation^[^ ^65^, ^66^, ^67^ ^]^	Not determined
Neurotrophic factors (NGF, BDNF, NT‐3) and glial‐derived neuroprotective peptides (NAP, ADNF‐9)	Activation^[^ ^68^ ^]^	These factors or peptides may be involved in neuronal or glial protection, neural regeneration, remyelination, and immunosuppression^[^ ^69^ ^]^
Nicotinamide adenine dinucleotide (NAD^+^)	Activation^[^ ^70^ ^]^	Diminished serum NAD^+^ levels are observed in MS patients.^[^ ^71^ ^]^ NAD^+^ protects against axonal degeneration and demyelination in EAE animal models^[^ ^72^ ^]^
Nicotinamide mononucleotide adenylyltransferases (NMNATs)	Activation^[^ ^73^, ^74^ ^]^	NMNATs are survival factors of heathy axons^[^ ^75^ ^]^ and injured axons,^[^ ^72^, ^76^ ^]^ and exert neuroprotective effects^[^ ^77^ ^]^
Cholesterol Breakdown Products	Activation^[^ ^14^, ^78^ ^]^	The level of cholesterol metabolites is increased in MS patients, and was proposed to activate PARP1 activity in neural ad immune cells of MS patients and animal models.
Sirtuins (SIRTs) – NAD^+^‐dependent deacetylase	Inhibition^[^ ^79^ ^]^	Sirtuin 1 (SIRT1) activation prevents axonal degeneration^[^ ^76^ ^]^ and exerts anti‐inflammatory effects.^[^ ^80^ ^]^
		Sirtuin 2 (SIRT2) is an oligodendrocyte‐specific protein and may modulate oligodendrocyte differentiation and myelination^[^ ^81^ ^]^
Histone variant macroH2A1.1	Inhibition^[^ ^43^, ^82^ ^]^	Not determined

_**Abbreviations**: NGF, nerve growth factor; BDNF, brain derived neurotrophic factor_ _; Neurotrophin‐3, NT‐3; NAPVSIPQ, NAP; ADNF‐9, ADNF‐9, activity dependent neurotropic factor 9._

Protein PARylation is a reversible and highly dynamic process. The in vivo half‐life of PAR chains is within several minutes.^[^ ^45^ ^]^ The rapid PAR turnover is catalyzed primarily by the PAR degrading (de‐PARylation) enzyme PAR glycohydrolase (PARG) and terminal ADP‐ribose glycohydrolase (de‐MARylation) enzyme TARG (**Figure** 1A). There are a growing number of pharmacological inhibitors identified which target PARP1 and PARG (**Figure** 1A). These inhibitors have been used for exploring the therapeutic potentials and mechanistic actions of PARP1‐mediated PARylation in various cancer cells and neurological conditions including MS as discussed in the following sections.

## 3 PARP1‐Mediated Cell Survival or Cell Death: Context‐Dependent Cell Fate Choice

**Figure 2:**
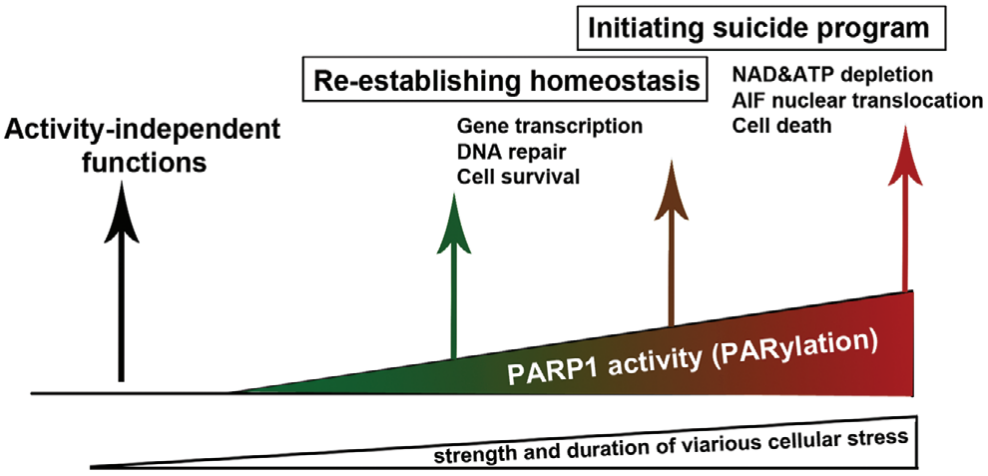
Context‐dependent outcomes of PARP1 and its activity.

PARP1‐mediated signaling has been proposed as an adaptive mechanism in response to various types of stress signals, for example, hormonal, inflammatory, oxidative, and nitrosative stress.^[^ ^46^ ^]^ Under physiological conditions, PARP1 regulates basic molecular and cellular processes through its activity‐independent and dependent functions (**Figure** **2**). For example, PARP1 plays a crucial role in stem cell maintenance and differentiation by modulating the function of the stem cell factor SRY‐Box transcription factor 2 (SOX2).^[^ ^34^, ^47^ ^]^ In the murine brain, PARP1 deletion promotes neural stem cells (NSCs) in the subventricular zone towards an oligodendroglial fate,^[^ ^48^ ^]^ suggesting a crucial role of PARP1 in NSC maintenance.

Historically, DNA strand breaks are the first identified trigger for PARP1 activation. Therefore the first well‐appreciated function of PARP1 is to mediate DNA damage response and repair.^[^ ^49^ ^]^ Indeed, PARP1‐deficient cells and mice are more sensitive than wild‐type counterparts to genotoxic agents.^[^ ^50^ ^]^ Seminal studies in 2005^[^ ^51^ ^]^ demonstrated a specific lethality of homologous recombination‐deficient cancer cells upon PARP1 inhibition, paving the way to exploiting its DNA repair function for clinical cancer therapy.^[^ ^3^ ^]^ The anti‐cancer effect of PARP1 inhibition supports PARP1‐mediated signaling as an endogenous protective mechanism in response to DNA damage. However, accumulating evidence has demonstrated that PARP1 could also be activated in a fashion independent of DNA damage under physiological conditions (Table 1). For example, neuronal activity, ERK1/2 signaling, neurotransmitters, neurotrophic factors, and NAD^+^ metabolism have been shown to activate PARP1 in various contexts. Some of these upstream PARP1 modulators are dysregulated in MS and EAE, suggesting that PARP1 activation observed in MS patients may be attributed to DNA damage and/or other factors independent of DNA damage.

Although PARP1 activation acts to re‐establish cellular homeostasis in response to mild‐to‐moderate stress signals, sustained and severe stress signals result in aberrant PARP1 hyperactivation which eventually leads to cell death (Figure 2). Consistent with this concept, high dose of the DNA toxic agents, *N*‐methyl‐*N*′‐nitro‐*N*‐nitrosoguanidine (MNNG) and hydrogen peroxide (H_2_O_2_) commonly used for studying cell death mechanisms, cause dysregulated PARP1 hyperactivity and ultimately lead to cell death including neuronal death.^[^ ^83^ ^]^ Mechanistically, PARP1‐mediated cell death (designated as parthanatos^[^ ^84^ ^]^) has been attributed to NAD^+^ depletion (consequently ATP depletion) which leads to necrotic cell death,^[^ ^85^ ^]^ release of mitochondrial protein apoptosis‐inducing factor (AIF) which leads to caspase‐independent apoptotic cell death,^[^ ^83c^ ^]^ and formation of excessive toxic PAR polymers.^[^ ^83b^ ^]^ At the molecular level, the binding of PAR polymers to AIF^[^ ^83a^ ^]^ is essential and sufficient for macrophage migration inhibitory factor (MIF)‐mediated DNA fragmentation during PARP1‐mediated cell death.^[^ ^86^ ^]^ Interestingly, caspase‐mediated PARP1 cleavage into 89 and 24 kDa fragments is a hallmark of ATP‐dependent apoptotic cell death.^[^ ^87^ ^]^ But both PARP1 and PARP1 cleavage are dispensable for caspase‐mediated apoptosis.^[^ ^88^ ^]^ There is some evidence suggesting that NAD^+^ depletion is not sufficient for PARP1 hyperactivation‐elicited neuronal death.^[^ ^84^ ^]^ Instead, PAR‐dependent inhibition of glycolysis, which precedes NAD^+^ depletion, accounts for PARP1 hyperactivation‐elicited cortical neuron death in vitro.^[^ ^89^ ^]^ Therefore, PARP1 hyperactivation results in NAD^+^ depletion and energy deficit, but energy deficit is independent of NAD^+^ depletion at least in primary cortical neurons.

Previous studies reported that PARP1 activation is required for primary neuron death elicited by peroxynitrite,^[^ ^55^ ^]^ a DNA toxic agent triggering nitrosative stress in MS patients,^[^ ^90^ ^]^ but it is dispensable for primary OL death induced by peroxynitrite.^[^ ^91^ ^]^ These discrepant observations underscore a cell type‐dependent or context‐dependent role of PARP1 activation in cell death. Taken together, PARP1‐mediated signaling itself represents an endogenous defense mechanism in response to physiological cues and pathological stresses. The fate choice of PARP1 in determining cell survival versus cell death depends on the cell type, the cellular stress type, and the strength of cellular stress (Figure 2).

## 4 Role of PARP1 in EAE Disease Severity and Pathology

**Figure 3:**
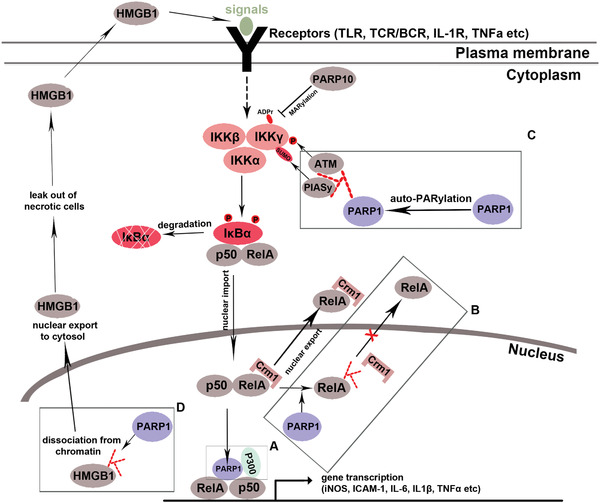
NF‐*κ*B signaling and its regulation by PARP1 and ADP‐ribosylation. In the classical (or canonical) pathway (depicted here),^[^ ^93^ ^]^ binding of extracellular signals to the membrane receptors leads to activation of the IκB kinase (IKK) complex (consisting two catalytic subunits IKK*α* and IKK*β* and one inhibitory subunit IKK‐*γ*, aka NF‐kappa‐B essential modulator (NEMO)), which subsequently phosphorylates I*κ*B*α* and facilitates dissociation of I*κ*B*α* from RelA(p65)/p50 complex. RelA/p50 heterodimer is then imported into the nucleus, binds to the responsive elements located at NF‐kB target gene promoters, and activates target gene transcription. Crm1‐mediated RelA (a.k.a. p65) nuclear export facilitates the cytoplasmic retention of RelA, thus downregulating NF‐kB target gene expression. PARP1 has been reported to regulate NF‐kB through multiple mechanisms in a context‐dependent manner (Boxes A–D). Box A: In LPS or TNF*α*‐stimulated primary mouse embryonic fibroblasts (MEFs) and HeLa cells, PARP1 augments NF‐kB‐mediated gene expression by directly interacting with RelA/p50 and histone acetyltransferase P300, a process independent of PARP1's catalytic activity or DNA binding function,^[^ ^94^ ^]^ Box B: In LPS‐stimulated smooth muscle cells (SMCs), PARP1 PARylates RelA and PARylated RelA prevents the binding to Crm1, leading to the nuclear retention of RelA and enhancing NF‐kB‐mediated gene expression.^[^ ^38^ ^]^ Oddly, in smooth muscle cells (SMCs) stimulated by TNF*α*, RelA nuclear trafficking is not altered by PARP1 inhibition,^[^ ^95^ ^]^ suggesting a context‐dependent regulation of NK‐kB signaling by PARP1. Box C: In mouse embryonic fibroblasts (MEFs) stimulated by DNA‐damaging dose of irradiation, DNA breaks induce PARP1 activation and PARP1 auto‐PARylation (auto‐modification). Auto‐PARylated PARP1 recruits IKK*γ*, PIAS*γ*, and ataxia‐telangiectasia mutated (ATM) to form a “signalosome” in which PAIS*γ* and ATM SUMoylates and phosphorylates IKK*γ*, respectively, two essential events for IKK kinase activation and, subsequently, the cascade of NF‐kB activation.^[^ ^96^ ^]^ Box D: In MEFs stimulated by DNA alkylating agents MNNG, nuclear PARP1 catalyzes PARylation of high mobility group box 1 (HMGB1), a chromatin‐binding protein and a robust trigger of innate immune activation. HMGB1 PARylation facilitates its dissociation from chromatin and promotes its nucleus‐to‐cytoplasm shuttle.^[^ ^97^ ^]^ Cytoplasmic HMGB1 may lead out of necrotic cells into the extracellular space where it may bind to Toll‐like receptors (TLRs) and further augment the downstream NF‐*κ*B signaling. Interestingly, in immortalized HeLa and U2OS cells stimulated by IL‐1*β* and TNF*α*, the mono‐ADP‐ribosylation enzyme PARP10 MARylates IKK*γ* and the MARylation event prevents the activation of IKK complex, thus downregulating NF‐kB cascade activation. Hence PARP10 antagonizes NF‐kB.^[^ ^98^ ^]^

Shortly after PARP1‐KO transgenic lines were generated^[^ ^50^, ^88^ ^]^ for studying DNA repair and cell survival, a seminal study reported that PARP1‐KO mice are extremely resistant to lipopolysaccharides (LPS)‐induced endotoxic shock through a defective nuclear factor *κ*B (NF‐*κ*B) activation.^[^ ^92^ ^]^ This study opened a new research direction linking PARP1 and NF‐*κ*B in vivo. Subsequent studies have suggested that PARP1 regulates NF‐*κ*B‐mediated signaling pathway by multiple mechanisms (**Figure** **3**). Because NF‐*κ*B is a master transcription factor for neuroinflammation in MS and EAE,^[^ ^93^ ^]^ a growing number of studies have been dedicated to exploring the effect of inhibiting PARP1 activity on the disease course and pathology of MS patients and EAE animals.

### 4.1 EAE Outcomes of Pharmacological PARP Inhibition and Genetic PARP Deficiency

Earlier studies explored the role of PARP1 in EAE by applying a variety of small inhibitors of PARP1 to different mouse models of EAE and assessing immune activation and response (**Table** **2**). The overall conclusion from these pharmacological studies is that PARP1 inhibition is protective against EAE disease, likely through dampening peripheral immune activation and neuroinflammation (Table 2). It remains enigmatic whether PARP1 activity in the CNS resident cells (neural and vascular cells) plays a role in regulating EAE disease course and pathology.

**Table 2:** Role of PARP1 inhibitors in EAE mouse models

PARP1 inhibitors	Animal models	EAE modulation	Pathology alterations	Ref.
PJ34 (20 mg kg^−1^, i.p.) or PHE (20 mg kg^−1^, i.p.) Twice a day from day 0 to day 12	MOG_35‐55_‐EAE in C57BL/6J mice (female)	Resistance to EAE assessed up to day 12	Reduced dendritic cell (DC) infiltration in the spinal cord in PJ34 (or PHE)‐treated mice	^[^ ^99^ ^]^
PJ34 (20 mg kg^−1^, i.p.) or PHE (20 mg kg^−1^, i.p.) Twice daily from day 1 to day 16 (disease initiation) or from day 22 to day 34 (disease relapse)	PLP_139‐151_‐EAE in SJL mice (female)	Reduced clinical score of the disease initiation and of the disease relapse	Reduced inflammatory infiltrations and demyelination during the disease initiation; Reduced T cell number and Th17 cell number in spinal cord during the disease relapse	^[^ ^99^ ^]^
PJ34 (10 mg kg^−1^ oral) Twice daily from day 7 through the terminal sacrifice at day 22	MBP‐EAE in SJL mice (female)	Reduced EAE incidence, mortality, and severity	Reduced mRNA levels of CD4, CD8, CD11b, and CD68, interferon gamma (IFN‐γ), inducible nitric oxide synthase (iNOS), TNFa, intercellular adhesion molecule 1 (ICAM‐1) genes in the spinal cord; Reduced blood brain barrier (BBB) permeability	^[^ ^100^ ^]^
5‐AIQ (3 mg kg^−1^ i.p.) Daily from day 20 through the terminal sacrifice at day 60.	MOG_35‐55_‐EAE in nonobese diabetic (NOD) mice ‐ secondary progressive EAE^#^	Reduced severity during the progressive phase of EAE	Reduced demyelination and axonal loss, Reduced density of IBA1^+^ cells (microglia and macrophages) and GFAP^+^ astrocytes.	^[^ ^14^ ^]^
Olaparib	A mouse model of localized neuroinflammation elicited by intracerebral injection of TNF*α*	Not applicable	Diminished BBB permeability; Reduced leukocyte migration across the BBB; decreased neuroinflammation. These findings indicate that PARP1 inhibition may maintain BBB integrity in MS and/or EAE.	^[^ ^101^ ^]^
Veliparib Rucaparib, Talazoparib	A mouse model of Parkinson's disease elicited by intrastriatal injection of *α*‐synuclein preformed fibrils	Not applicable	Decreased dopamine neuronal loss. The finding indicates that PARP1 inhibition may protect neurons from damage during the time course of MS and/or EAE.	^[^ ^12b^ ^]^
Olaparib (0.1 nM–10 µM)	In vitro oligodendrocyte culture	Not applicable	Olaparib induces OPC death and inhibits OPC differentiation into oligodendrocytes in the dish. These findings indicate that PARP1 inhibitors may exert a detrimental effect on oligodendrocyte survival and myelin repair in MS and/or EAE	^[^ ^102^ ^]^

^#^ a subsequent study demonstrated that MOG_35‐55_‐EAE of nonobese diabetic (NOD) mice is not a progressive EAE model and that the seemingly progressive course seen in clinical score of MOG_35‐55_‐EAE NOD mice is likely an artifact of data handling and interpretation^[^ ^21^ ^]^

In stark contrast to pharmacological inhibition, several studies including the one from our group^[^ ^22b^ ^]^ employed PARP1‐KO mice reporting discrepant or even opposite results (**Table** **3**). PARP1‐KO mice displayed earlier onset and more severe peak clinical score than PARP1‐WT littermate controls when challenged with MOG_35‐55_‐EAE.^[^ ^22b^ ^]^ The increased EAE clinical scores were confirmed by a separate study from an independent group reporting that PARP1 deficiency was not protective but rather enhanced peak disease severity of MOG_35‐55_‐EAE‐injured PARP1‐KO mice compared to PARP1‐WT controls.^[^ ^103^ ^]^ While 15*α*‐hydroxichelestene (15‐HC), a cholesterol oxidation product, worsened disease severity of MOG_35‐55_‐EAE presumably through PARP1's catalytic functions,^[^ ^14^ ^]^ PARP1 deficiency, per se, neither protected against MOG_35‐55_‐EAE (see **Figure** **4**C of ref. [14]), nor reduced the mRNA levels of iNOS, TNF*α*, and C‐C motif chemokine 2 (CCL2) (NF‐kB target genes and pro‐inflammatory mediators) in CD11b^+^ cells in the spinal cord (see **Figure 4E** of ref. [14]). Together, unlike pharmacological PARP1 inhibition, genetic PARP1 deficiency aggravates the disease severity of EAE, suggesting that PARP1 confers protection against inflammatory demyelinating insults and that the hypothetical protective effect may extend beyond immunomodulation.

**Figure 4:**
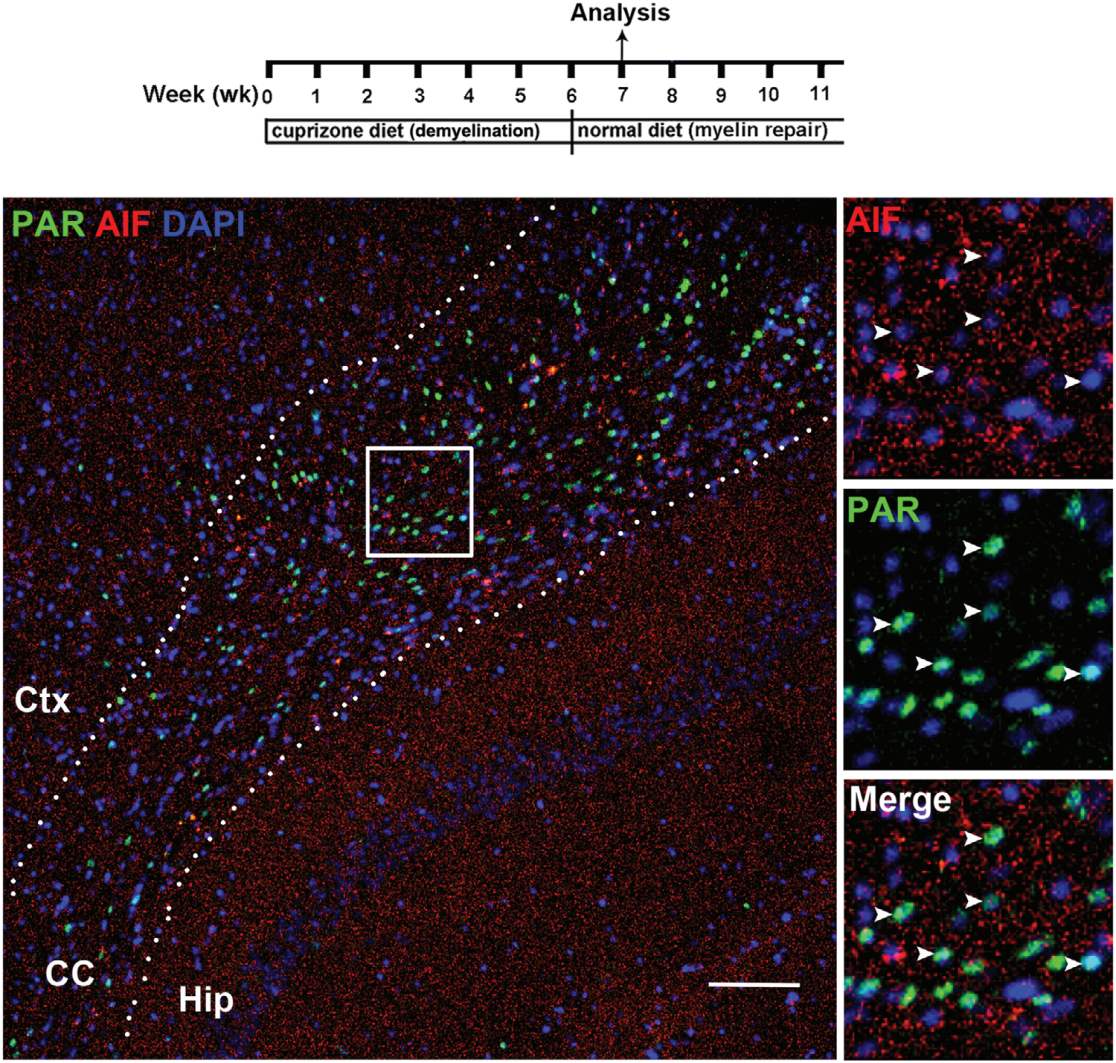
Double immunohistochemistry (IHC) of PAR and SOX10 (A) and of PAR and AIF (B) in the brain corpus callosum (CC) of mice. A) nuclear PAR signal (co‐labeled with the nuclear dye DAPI) is mutually exclusive from SOX10^+^ oligodendroglial lineage cells during the demyelination stage at 3 weeks of cuprizone diet. Insert A1 is IHC of CD68, a marker of activated microglia, on a consecutive section adjacent to (A), showing that PAR^+^ area is correlated with CD68^+^ area in the corpus callosum. B) numerous PAR^+^ signals are mutually exclusive from AIF in nucleus during the remyelination stages at 1 week after returning to the normal diet. Note the punctate staining pattern of mitochondrial AIF protein and no evidence of AIF in the nucleus. Arrowheads point to PAR^+^ cells. Boxed areas in (A, B) are shown at higher magnification at the right. Ctx, cortex; Hip, hippocampus. Dotted lines delineate the corpus callosum. Scale bar = 100 µm.

**Table 3:** Role of PARP1 depletion in EAE mouse models

PARP1 genetic mice	MS models, genetic background	EAE severity (KO vs WT mice)	Pathological alterations (KO vs WT mice)	Ref.
PARP1‐KO (129S background)	MOG_35‐55_‐EAE in 129S mice (*n* = 30 PARP1‐WT and 30 PARP1‐KO), disease monitored throughout day 35	Increased incidence, early onset, and increased severity on days 11–17	Increased CNS infiltration of CD4^+^ T cells at day 10. Increased number in CNS macroglia and macrophages at day 10. No changes in the number of peripheral T lymphocytes, dendritic cells, or macrophages	^[^ ^22b^ ^]^
PARP1‐KO (129S background)	MOG_35‐55_‐EAE in 129S mice (*n* = 5 PARP1‐WT, 9 PARP1‐KO), disease monitored throughout day 17	Statistics not determined Peak clinical score (mean ± s.e.m.): PARP1‐WT:2.2 ± 0.2 PARP1‐KO:1.61 ± 0.7	No changes in the mRNA levels of CCL2, iNOS, TNF*α* in CD11b^+^ cells isolated from the spinal cord of EAE	^[^ ^14^ ^]^
PARP1‐KO (129S background)	MOG_35‐55_‐EAE in 129S background mice	The authors described consistent results as those reported by Selvaraj et al., 2009^[^ ^22b^ ^]^ but did not show the data	Not determined	^[^ ^103^ ^]^
PARP2‐KO (C57BL/6)	MOG_35‐55_‐EAE in C57BL6/J mice (*n* = 9 PARP2‐WT and 6 PARP2‐KO) disease monitored throughout day 35	Delayed onset and reduced severity of EAE	Reduced number of spinal CD4^+^ T cells and Th1 and Th17 T cell subpopulations (by immunohistochemistry) Reduced demyelination at peak EAE disease (by immunohistochemistry) Reduced number of spinal CD11b^+^ macrophages and microglia (by immunohistochemistry)	^[^ ^103^ ^]^

### 4.2 Strengths and Limitations of PARP1 Pharmacological Inhibition and Genetic Deficiency in EAE Studies and Possible Interpretations

The reasons for the differential effects between pharmacological PARP1 inhibition and genetic PARP1 deficiency on EAE disease severity are unclear, and limited data are available to directly interpret the discrepancy. The following aspects cannot be neglected for interpreting the discrepant observations.

First, while pharmacological PARP1 inhibition is advantageous over genetic deficiency for the purpose of clinical translation, it is difficult to attribute the observed biological effects solely to PARP1 inhibition. The observed effect of PARP1 inhibitors on EAE disease may also result from inhibition of PARP1‐unrelated targets or molecular pathways.^[^ ^104^ ^]^ A study in 2012 comprehensively analyzed the binding specificity of 185 known and potential PARP inhibitors with 13 of the 17 human PARP family members. The authors in that study concluded that most inhibitors including those which are commonly used in basic/preclinical research and patient care have the binding ability to not only PARylation enzymes, PARP1, PARP2, PARP5a (Tankyrase 1), and PARP5b (Tankyrase 2), but also to MARylation enzymes PARP3, PARP4, PARP10, PARP14, PARP15, and PARP16 albeit with differential affinity.^[^ ^105^ ^]^ PJ34, a potent PARP1 inhibitor that had been used in studying diabetic pathology^[^ ^106^ ^]^ and later in MS (Table 1) and other physiological and pathological conditions in over 300 publications, was shown to inhibit PARP1, PARP2, PARP3, PARP4, PARP5a, PARP5b, PARP10, PARP14, and PARP15.^[^ ^105^ ^]^ In addition, PJ34 was reported to inhibit non‐PARP members, for instance, the inflammation modulators metalloproteinase 9 (MMP9),^[^ ^107^ ^]^ MMP2,^[^ ^108^ ^]^ PIM serine/threonine kinase family proteins PIM1/PIM2,^[^ ^109^ ^]^ and likely P2Y12 receptor signaling.^[^ ^110^ ^]^ A growing number of studies have demonstrated a crucial pathophysiological role in MS and EAE for MMP2,^[^ ^111^ ^]^ MMP9,^[^ ^112^ ^]^ and P2Y12 receptor signaling^[^ ^113^ ^]^ and revealed an anti‐inflammatory effect of PIM1/2 inhibition through suppressing NF‐*κ*B signaling.^[^ ^114^ ^]^ Corroborating this idea, all publications administering PJ34 in the presence of PARP1 KO observed PARP1‐independent effects.^[^ ^109^ ^]^ The promiscuous inhibition of a growing number of off‐targets by PJ34 makes it difficult to interpret its biological effects on MS and EAE. Moreover, 5‐AIQ and PHE previously used in EAE studies inhibit not only PARP1‐3, but also Tankyrases with approximately equal potency.^[^ ^105^ ^]^ Tankyrase inhibition has been demonstrated to antagonize the activity of Wnt/*β*‐catenin signaling^[^ ^115^ ^]^ which plays a context‐dependent role in regulating inflammation,^[^ ^116^ ^]^ oligodendrogliopathy,^[^ ^117^ ^]^ and neuropathy/axonopathy.^[^ ^118^ ^]^ Off‐target effects are also observed for FDA‐approved the third generation PARP inhibitors^[^ ^119^ ^]^ which were reported to be effective in reducing neuroinflammation and neuronal damage in relevant preclinical animal models (Table 2). These potential and demonstrated off‐target effects were largely neglected for data interpretation in previous publications.

Second, while the observed reduction of immune reaction in EAE mice by PARP1 inhibitors was interpreted to be a result of suppressing PARP1‐activated NF‐*κ*B signaling (Figure 3), it is also possible that the immune reduction may result from the death of immune cells elicited by PARP1 inhibitors which have been shown to kill tumor cells via the additional PARP1‐trapping mechanism.^[^ ^120^ ^]^ Peripheral lymphocytes and dendritic cells (DCs) are highly proliferative and significantly expanded in numbers prior to infiltrating into the CNS of EAE mice.^[^ ^22b^ ^]^ Highly proliferative cells are prone to generate DNA breaks during replication^[^ ^121^ ^]^ which could recruit and activate PARP1. It has been shown that PARP1 inhibitors cause PARP1 trapping at damaged DNA^[^ ^122^ ^]^ and that trapped PARP1‐DNA complexes are more cytotoxic than unrepaired DNA breaks caused by PARP1 inactivation.^[^ ^123^ ^]^ It is plausible that the reduced CNS infiltration of lymphocytes and DCs reported in previous pharmacological studies (Table 2) may simply attribute to the death of peripheral immune cells elicited by PARP1 inhibitors. To support this possibility, PARP1 inhibitors were reported to reduce the number of peripheral DCs in the lymphoid organs of immunogen‐challenged mice.^[^ ^100^ ^]^ In contrast to PARP1 inhibitors, however, PARP1 trapping is unlikely to occur in genetic PARP1 deficient mice given the lack of PARP1 molecules. This may explain the comparable number of myelin‐specific T cells and DCs in the spleen of MOG_35‐55_‐immunization between PARP1‐KO and PARP1‐WT mice prior to EAE disease onset.^[^ ^22b^ ^]^

Third, as demonstrated in the previous study,^[^ ^105^ ^]^ most PARP1 inhibitors could dampen the activity of not only PARP1 but also other PARP members. It is possible that PARP family members may exert different effects on the disease course of MS and EAE. Animal data using genetic PARP1‐KO and PARP2‐KO mice have shown opposite effects of PARP1 and PARP2 on EAE disease severity: PARP1 is protective yet PARP2 is detrimental (Table 2). Therefore, potential compensatory effects and/or opposite roles of different PARP members may partially account for the discrepancy effect of PARP inhibition and PARP1 KO on EAE disease severity.

Genetic PARP1 deficiency approach, however, circumvents the issue of promiscuous inhibition of PARP members and non‐PARP off‐targets by PARP1 chemical inhibitors. We and others demonstrated that PARP1 depletion did not alter the expression of major proinflammatory cytokines nor canonical NF‐*κ*B target gene expression in the spinal cord^[^ ^22b^ ^]^ or in CD11b^+^ cells (macrophages, microglial, and neutrophils) sorted from the spinal cord^[^ ^14^ ^]^ during the acute inflammatory phase of MOG_35‐55_‐EAE. Caution should also be used, however, for interpreting the data collected from germline PARP1‐deficient mice (Table 3). Although PARP1‐KO mice are viable and fertile, likely due to functional overlap of PARP1 and PARP2,^[^ ^124^ ^]^ they display many developmental defects at cellular levels such as affected circulating immune cell profiles in the blood,^[^ ^22b^ ^]^ increased T cell numbers in the peripheral lymphoid organs^[^ ^22^, ^125^ ^]^ including T regulatory cells (Tregs),^[^ ^126^ ^]^ and compromised NSC functions.^[^ ^48^, ^127^ ^]^ The differences in the physiological immune cell profiles and NSCs may confer differential susceptibility or resistance to EAE disease between PARP1‐WT and KO mice. Given PARP1 activation in multiple lineage cells of MS patients and EAE animal models including T and B lymphocytes, monocytes, DCs, macrophages, microglia, OLs, astrocytes, and neurons,^[^ ^13^, ^14^, ^15^, ^16^ ^]^ the biological effect of PARP1 deficiency/inhibition on different types of immune and neural cells cannot be neglected prior to considering PARP1 as a potential therapeutic target for MS or EAE. Hence, future studies are needed to dissect the cell‐dependent role of PARP1 in pathogenesis and neurological progression of MS and EAE. In this regard, recently engineered *Parp1*‐floxed mice^[^ ^40^ ^]^ could help define cell type‐specific role of PARP1 in EAE pathogenesis and disease regulation by leveraging the in vivo conditional KO of *Cre‐loxP* approach.^[^ ^128^ ^]^

## 5 Immunomodulatory Role of PARP1: Focusing on PARP1‐Deficiency Experimental Data

It is generally accepted that the initiation and relapses of MS are correlated with activation of peripheral immune cells and their subsequent infiltration into the CNS.^[^ ^129^ ^]^ The role of PARP1 and its PARylation activity in peripheral immune activation has been extensively studied and the prevailing conclusion is that PARP1 promotes immune activation despite inconsistent observations have been made in previous studies using pharmacological and genetic approaches under different settings. As critically assessed above, most PARP inhibitors have apparently different off‐target effects; we review PARP1‐mediated immunomodulation with a particular focus on studies using PARP1‐deficient experimental systems.

### 5.1 PARP1 in CD4^+^ T Cell Activation and Polarization

The activation of CD4^+^ autoreactive T cells and their polarization (aka differentiation) into different mature effector cells are essential for inflammatory CNS attacks and the disease progression of MS and EAE. The nuclear factor of activated T cells (NFAT) family of transcription factors, which share a conserved DNA‐binding domain structurally related to the REL‐homology domain of NF‐*κ*B family members, are essential for T cell function and activation.^[^ ^130^ ^]^ Nuclear factor of activated T‐cells (NFAT) plays an essential role in EAE pathogenesis, as NFAT1 and/or NFAT2‐deficiency is protective against MOG_35‐55_‐EAE disease initiation and severity in mice.^[^ ^131^ ^]^ PARP1 directly interacts with NFAT in the nucleus of activated T cells where PARP1‐mediated PARylation increases the DNA binding affinity of NFAT and activates NFAT target gene expression, thus PARP1 PARylation activity contributing to NFAT‐dependent gene expression during CD4^+^ T cell activation.^[^ ^132^ ^]^ Consistently, the production of interleukin 2 (IL2), a canonical NFAT target gene,^[^ ^133^ ^]^ was significantly downregulated in PARP1‐deficient naïve CD4^+^ T cells stimulated by anti‐mouse CD3 and anti‐mouse CD28 in vitro.^[^ ^132^ ^]^ Oddly, in a separate study, Valdor et al., reported that PARP1 inhibitors increased IL2 gene expression in activated primary T cells and the mechanism was proposed through suppressing NFAT nuclear retention and transcription activity mediated by PARylation.^[^ ^134^ ^]^ It remains unclear why NFAT PARylation by PARP1 leads to the opposite biological outcomes of NFAT transcriptional activity in activated T cells. It is possible that the in vitro culture systems and/or approaches for PARP1 manipulation and T cell stimulation account for the discrepancies.

Once activated, CD4^+^ T cell is polarized into different subpopulations of T effector cells, such as Th1, Th17, Th2, or T regulatory cells Tregs, and contributes to pathogenesis of MS and EAE.^[^ ^135^ ^]^ Th1 and Th17 are generally thought to exert pro‐inflammatory effect while Th2 and Tregs anti‐inflammatory during the disease course of MS and EAE. Previous studies reported discrepant observations regarding the role of PARP1 in Treg cell polarization and function under different in vitro conditions. Nasta et al. reported that PARP1‐deficiency enhanced the differentiation (but not the immunosuppressive function) of Tregs (defined as CD4^+^CD25^+^Foxp3^+^) from naïve CD4^+^ T cells stimulated by anti‐mouse CD3/CD28 mAbs,^[^ ^126a^ ^]^ which was confirmed by a subsequent study.^[^ ^126b^ ^]^ Mechanistically, PARP1 activity in T cells was proposed as an inhibitor for transforming growth factor‐β (TGF*β*) receptor‐mediated signaling,^[^ ^126b^ ^]^ an essential pathway regulating Treg differentiation.^[^ ^136^ ^]^ Regarding the role of PARP1 in Treg cell function, in contrast to a report by Nasta et al.,^[^ ^126a^ ^]^ a later study showed that PARP1 inhibition by pharmacological compounds augmented the immunosuppressive function of human primary Tregs stimulated by anti‐CD3/CD28 mAbs, as assessed by in vitro proliferation inhibition assay,^[^ ^137^ ^]^ suggesting that PARP1 activity suppresses the inhibitory function of Tregs, a conclusion in line with a study using PARP1‐KO assay.^[^ ^138^ ^]^

PARP1 may also control Th1/Th2 balance under stimulation conditions. Microarray analysis demonstrated that, in naïve CD4^+^ T cells stimulated by anti‐CD3/CD28 mAbs in vitro, PARP1 deficiency increased the expression of Th1 signature cytokine IFN‐*γ* and decreased Th2 signature cytokine IL‐4,^[^ ^139^ ^]^ suggesting that PARP1 may control the Th1/Th2 balance. Consistently, later data demonstrated that, compared to PARP1‐intact cells, PARP1‐deficient naïve CD4^+^ T cells from the spleen generated a decreased frequency of Th2 cells expressing IL4, IL5, and GATA binding protein 3 (GATA‐3) under both nonskewing and Th2‐skewing culture conditions without affecting Th1 cell differentiation,^[^ ^140^ ^]^ indicating that PARP1 promotes Th2 yet is dispensable for Th1 cell differentiation in vitro.

IL17‐producing CD4^+^ T effector cells (Th17 cells) play a pivotal role in the pathogenesis of MS and EAE.^[^ ^141^ ^]^ PARP1 has been reported in some but not all studies to regulate Th17 cell differentiation. Earlier data demonstrated that PARP1 ablation did not affect naïve CD4^+^ T cell differentiation into Th17 cells nor the expression of IL17 and ROR*γ* (an essential transcription factor for Th17 differentiation) in Th17‐skewing conditions (TGF*β*1+IL6),^[^ ^126a^ ^]^ suggesting a dispensable role of PARP1 in Th17 differentiation. However, a later study reported a more robust differentiation of Th17 and production of more IL17 in PARP1‐deficient T cells than in PARP1‐intact T cells in Th17‐skewing conditions (TGF*β*1+IL‐6),^[^ ^126b^ ^]^ indicating a repressive role of PARP1 in Th17 cell differentiation. In the context of EAE, we found that PARP1 deficiency did not affect the number of INF*γ*‐producing Th1 and IL17‐producing Th17 cells in the spleen and lymph nodes of PARP1‐KO mice compared to PARP1‐WT animals in response to MOG_35‐55_‐EAE.^[^ ^22b^ ^]^ These data suggest that PARP1 plays a dispensable role in Th1/Th17 polarization from CD4^+^ T cells. It is possible that another PARylation enzyme, PARP2, may compensate for PARP1 deficiency in regulating Th1/Th17 polarization in vivo. Interestingly, in contrast to PARP1‐KO mice,^[^ ^22b^ ^]^ PARP2‐KO mice exhibited a significant reduction in overall MOG_35‐55_‐EAE disease and peak neurological dysfunction,^[^ ^103^ ^]^ suggesting that PARP1 and PARP2 may antagonize each other in EAE disease pathogenesis or in Th1/Th17 cell polarization if any. Future EAE studies using CD4^+^ T cell‐specific PARP1‐KO/PARP2‐KO may help prove or disprove the potential functional redundancy or antagonism in regulating CD4^+^ T cell polarization in vivo.

### 5.2 PARP1 in DCs and Macrophages

Antigen‐presenting cells (APCs) are essential for T cell polarization and inflammation.^[^ ^142^ ^]^ DCs and macrophages (monocyte‐derived macrophages and CNS‐resident perivascular macrophages, PVMs, or activated microglia) are the primary APCs that play an essential role in MS and EAE. The role of PARP1 in DC migration, differentiation, and function is still controversial. In vitro pharmacological data demonstrated that chemical‐elicited PARPs inhibition reduced DC differentiation and maturation from peripheral blood mononuclear cells (PBMCs)^[^ ^143^ ^]^ and prevented CNS migration of DCs from the peripheral secondary lymphoid organs in a mouse model of ovalbumin (OVA) immunization and in the setting of murine EAE injury.^[^ ^99^ ^]^ However, genetic data showed that PARP1 deficiency did not affect in vitro DC differentiation, maturation, or function of PARP1‐KO PBMCs,^[^ ^144^ ^]^ underscoring the different conclusions from genetic depletion versus pharmacological inhibition in DC biological outcomes. Interestingly, PARP1 genetic depletion was reported to reduce DC migration to the lungs but not to the spleens and lymph nodes of OVA‐immunized PARP1‐KO mice,^[^ ^144^ ^]^ suggesting a context‐dependent role of PARP1 in DC migration. In the setting of MOG_35‐55_‐EAE, our genetic data showed that PARP1 deficiency did not alter the number of total DCs and co‐stimulatory molecule CD80^+^/CD86^+^ DCs in PARP1‐KO mice nor the phagocytosis function of PARP1‐KO DCs,^[^ ^22b^ ^]^ suggesting that PARP1 is dispensable for DC functional maturation in EAE.

NF‐*κ*B is a central regulator for macrophage activation. It is generally believed that PARP1 promotes pro‐inflammatory macrophage polarization by activating NF‐*κ*B and its target genes IL1*β*, IL6, TNF*α*, and iNOS (see review^[^ ^145^ ^]^). While PARP1 activates NF‐*κ*B‐mediated pro‐inflammatory pathway (Figure 3), the anti‐inflammatory role of PARP1 is not uncommon. During inflammation, caspase‐mediated cleavage of PARP1 (consequently disrupting PARP1's catalytic function) is required for enhancing LPS‐stimulated activation of NF‐*κ*B and its target genes such as IL6, colony stimulating factor 2 (CSF2), and leukemia inhibitory factor^[^ ^146^ ^]^ as well as IL1*β* and TNF*α* ^[^ ^88a^ ^]^ in macrophage both in vitro and in vivo. These data suggest that PARP1 may repress NF‐*κ*B‐mediated pathways in response to proinflammatory stimuli. It was subsequently reported that PARP1 depletion augments LPS‐stimulated expression of IL6 in PARP1‐deficient bone marrow‐derived macrophages compared to PARP1‐intact counterparts,^[^ ^147^ ^]^ further supporting a role of PARP1 in antagonizing the proinflammatory function of NF‐*κ*B in macrophages. Mechanistically, the repressive role of PARP1 in IL6 expression is independent of its PARylation function yet depends on its chromatin‐binding function; PARP1 occupies the chromatin at the IL6 promoter in macrophages and interferes with methytransferase mixed lineage leukemia protein‐1 (MLL1)‐dependent histone H3 lysine 4 trimethylation (H3K4me3) at the IL6 promoter,^[^ ^147^ ^]^ an epigenetic modification that activates the transcription of nearby genes. More recently, PARP1 enzymatic activity was reported to mediate the anti‐inflammatory effect of resveratrol^[^ ^148^ ^]^ presumably by repressing the expression of pro‐inflammatory cytokines TNF*α* and IL6 in human monocytic cell line (THP‐1 cells)‐derived macrophages.^[^ ^149^ ^]^ In that study, nicotinamide was shown as an agonist for PARP1 activation in macrophages.^[^ ^148^ ^]^ Oddly, it is generally accepted that nicotinamide inhibits PARP1 activity in cells.^[^ ^150^ ^]^ Together, it is possible that PARylation‐dependent function of PARP1 promotes NF‐*κ*B‐mediated pro‐inflammatory pathway whereas PARylation‐independent function of PARP1 suppresses NF‐*κ*B‐mediated pro‐inflammatory pathways in macrophages and other immune cells. This context‐dependent hypothetic model may provide an explanation underlying the discrepant observations made with PARP1 inhibitors and genetic deficiency in EAE.

### 5.3 Cellular NAD^+^ Levels Link PARP1 Activity and IDO‐Kynurenine Pathway and Coordinate Neuroinflammation

NAD^+^ is the ADP‐ribose donor for PARylation and it is also a potent activator for PARP1.^[^ ^70^ ^]^ In mammalian cells, the de novo IDO‐kynurenine pathway generates NAD^+^ through catabolizing tryptophan (Figure 1A). The IDO‐kynurenine pathway is acutely activated in APCs (macrophages, microglia, and DCs) in response to Th1 signature cytokine interferon *γ* (IFN*γ*) and has been demonstrated to inhibit autoreactive T cell proliferation through extracellular tryptophan depletion,^[^ ^151^ ^]^ thus exerting a potent anti‐inflammatory effect. The increased end‐product NAD^+^ by IDO‐kynurenine pathway^[^ ^152^ ^]^ may favor PARP1 activation in APCs which promotes their activation, thus exerting a pro‐inflammatory effect.^[^ ^153^ ^]^ Therefore, PARP1 and IDO‐kynurenine pathway may coordinate neuroinflammation in MS and EAE. Furthermore, chronic activation of IDO‐kynurenine pathway produces excessive neurotoxic and gliotoxic metabolites and depletes NAD^+^ in neighboring neural cells,^[^ ^154^ ^]^ potentially causing inflammation and cytotoxicity. Hence, the effect of IDO‐kynurenine (or PARP1) on neuroinflammation and EAE may be complex and context‐dependent. Consistent with the concept, recent data suggest that the effect of inhibiting IDO‐kynurenine pathway on neuroinflammation and EAE symptoms depends on the timing of inhibition and the disease severity of animals.^[^ ^155^ ^]^

## 6 Context‐Dependent Role of PARP1 in Neuronal Damage and Axonal Degeneration: Implications in MS and EAE

Neuronal damage and axonal degeneration are the direct substrates for MS disease progression. Unlike immunomodulation, the direct demonstration of a role for PARP1 in neuropathy and axonopathy of MS and EAE is still lacking. Nevertheless, previous studies have shown that excessive PARP1 activation mediates acute neuronal death after focal cerebral ischemia,^[^ ^156^ ^]^ neurotrauma,^[^ ^10^ ^]^ and in response to reactive oxygen (nitrogen) species‐induced CNS injury^[^ ^157^ ^]^ and glutamate‐mediated excitotoxicity.^[^ ^83^, ^158^ ^]^ These published data provide some clues for future studies testing the function of PARP1 in neuronal/axonal pathology in the context of MS and EAE. In a non‐human primate MS model, the product of PARP1 enzymatic activity, PAR, was reported to be upregulated in scattered neurons near demyelinating plaques.^[^ ^16^ ^]^ It remains enigmatic, however, whether PARP1 and its activity promote neuronal damage or enhance neuronal survival in the setting of MS and EAE.

### 6.1 PARP1 in Neuronal Cell Death and Survival: Does Injury Severity Matter?

Chronic oxidative and nitrosative stress causes damage to proteins, lipids, and DNA, and plays an important role in the pathogenesis and tissue damage of MS and EAE.^[^ ^52^, ^159^ ^]^ PARP1 is activated in neurons upon exposure to free radicals nitric oxide (NO),^[^ ^160^ ^]^ peroxynitrite,^[^ ^161^ ^]^ hydrogen peroxide (H_2_O_2_),^[^ ^83c^ ^]^ DNA alkylating agents MNNG,^[^ ^83^, ^85^ ^]^ and excitotoxic agent *N*‐Methyl‐D‐aspartate (NMDA).^[^ ^83^, ^158^ ^]^ PARP1 activation was shown to mediate necrotic neuron death by these agents through NAD^+^ depletion and energy failure. This concept provides rationales for preclinical studies exploring the neuroprotective role of PARP1 inhibition in various neurological disorders and injuries.

In contrast to the well‐accepted concept of PARP1 activation in neuronal death, some studies have reported a dispensable or even a protective role of PARP1 in neuronal death under different contexts. The extent of primary cerebellar neuronal death elicited by peroxynitrite (100 µM dose) or neurotoxin 1‐methyl‐4‐phenylpyridine (MPP^+^) (50 µM)^[^ ^162^ ^]^ was unaffected by genetic PARP1 deletion in PARP1‐KO neurons compared to PARP1‐intact neurons.^[^ ^163^ ^]^ These in vitro data suggest that PARP1 may be dispensable in primary neuron death under certain contexts. As proposed and illustrated in Figure 2, PARP1 activity, per se, is an endogenous adaptive protective mechanism in response to various physiological cues and cellular stress. Under excessive DNA damage conditions, aberrant PARP1 activity leads to NAD^+^ depletion and initiates cell suicide through AIF‐mediated parthanatos. It is noteworthy that most prior studies employed high doses of DNA damaging agents in micro‐molar concentrations, such as MNNG (500 µM), H_2_O_2_ (500 µM), NMDA (500 µM),^[^ ^83c^ ^]^ and peroxynitrite (50–1000 µM),^[^ ^161^ ^]^ to study the role of PARP1 in cell death. Brief incubation of primary neurons with such high doses of chemicals was reported to cause greater than 90% of cell death within 12–24 h.^[^ ^83^, ^85^ ^]^ Hence, it is not surprising that excessive DNA damage under such contexts leads to sustained and aberrant activation of PARP1 and that inhibiting PARP1 could slow NAD^+^ consumption, energy failure, and subsequent neuron death. It is possible that PARP1 may protect neurons against DNA damage agents when applied with lower doses. Supporting this possibility, previous data have shown that PARP1 inhibition enhances or prevents primary cortical neurons death depending on the severity of oxidative stress elicited by H_2_O_2_ incubation.^[^ ^164^ ^]^ In that study, Diaz‐Hernandez and colleagues demonstrated that primary cortical neurons upregulated PARP1 activity and caused cellular NAD^+^ depletion by ≈100% and ≈50% within 1 h in response to 500 µM H_2_O_2_ (representing severe oxidative stress) and 50 µM H_2_O_2_ (mild‐to‐moderate oxidative stress), respectively. Consistent with the differential severity of H_2_O_2_‐induced oxidative stress, greater than 30% primary neurons died within 1 h in 500 µM H_2_O_2_‐treated group whereas less than 25% died up to 18 h in 50 µM H_2_O_2_‐treated group. These observations are in line with previous data showing that 500 µM NMDA and 500 µM MNNG elicited greater than 90% cell death of primary neurons within 12–24 h.^[^ ^83^, ^85^ ^]^ Surprisingly, PARP1 inhibition by chemical compounds or shRNA‐mediated knockdown aggravated primary neuron death in 50 µM H_2_O_2_‐treated group but protected primary neurons from death in 500 µM H_2_O_2_‐treated group.^[^ ^164^ ^]^ These data clearly suggest that PARP1 activation serves as an endogenous protective mechanism in neurons under mild to moderate oxidative stress whereas its aberrant hyperactivity under severe oxidative stress rapidly exhausts cellular NAD^+^ pool and initiates cellular suicide program (parthanatos) (Figure 2). PARP1 activation was neuroprotective against oxidative neuron injury elicited by disrupting the homeostasis of the endogenous antioxidant glutathione,^[^ ^164^ ^]^ which better reflects in vivo pathophysiology in MS^[^ ^165^ ^]^ and neurodegenerative disorders^[^ ^166^ ^]^ compared with primary neurons acutely insulted with high doses of DNA‐damaging agents in vitro. These data suggest that translation of PARP1 inhibition to the therapeutics for MS and other neurodegenerative disorders shall consider the protective role of PARP1 in response to chronic mild‐to‐moderate injury. In this regard, more studies are needed to directly define the role of PARP1 in neuron death and survival in the setting of MS and animal models.

### 6.2 PARP1 in Neuronal Cell Death and Survival: Does Sex Matter?

Previous data suggest that the role of PARP1 in neuron death or survival may depend on animal sex. Employing a mouse model of middle cerebral artery occlusion (MCAO), which is widely used for mimicking pathology of ischemia stroke, McCullough and colleagues reported that PARP1 deletion exacerbated cerebral damage (i.e., large infarct volume) in female PARP1‐KO mice but attenuated cerebral damage in male PARP1‐KO mice.^[^ ^167^ ^]^ These contrasting observations suggest that PARP1 activity may be neuroprotective in females while neurotoxic in males. Like PARP1‐KO animals, deletion of neuronal‐specific nitric oxide synthase (nNOS), which has been proposed to act upstream of PARP1 activation to induce neuronal death in MCAO mouse model,^[^ ^156^ ^]^ worsened and alleviated cerebral damage in nNOS‐KO female and male mice, respectively.^[^ ^167^ ^]^ The sex difference in PARP1‐mediated neuronal death and survival was further confirmed in a recent study.^[^ ^168^ ^]^ Together, these data indicate a sexual dimorphism of PARP1 in neuronal death and survival at least in the context of ischemic stroke: neuroprotective in females and neurotoxic in males. Retrospectively, it is notable that most prior publications reporting a cytotoxic role of PARP1 in neuronal death employed male mice for studying the role of PARP1 in cerebral ischemia,^[^ ^156^ ^]^ Parkinsonism,^[^ ^162^ ^]^ NMDA‐induced excitotoxicity,^[^ ^158^ ^]^ and traumatic brain injury.^[^ ^169^ ^]^ It remains to be defined, however, whether PARP1 deletion or pharmacological inhibition worsens neuropathy in female mice of these experimental models. It will also be interesting to determine whether PARP1's role in neuropathy/axonopathy, immune activation, and oligodendrogliopathy exhibits sex dimorphism in MS and animal models. These questions need to be solved prior to translating PARP1 inhibition into therapies for MS and other neurological disorders and injuries.

### 6.3 PARP1 in Axonal Damage and Regeneration

PARP1 is an abundant nuclear protein and its activity is largely restricted to the nucleus (Figure 1B). PAR signals observed in the cytoplasm of neurons or the processes of glial cells in EAE^[^ ^14^, ^16^ ^]^ are likely derived from the PARylation activity of Tankyrase 1 (PARP5a) or Tankyrase 2 (PARP5b) which is abundantly distributed in the cytosol, or from PARP1‐generated PAR that leak out of the nucleus. How PARP1's nuclear activity affects the damage or survival of axons remains to be defined and likely acts through controlling neuronal damage or survival. Most prior studies focused on the role of PARP1 in axonal regeneration rather than degeneration. Using an in vitro model of axonal growth inhibition elicited by myelin‐associated protein (MAG), myelin‐derived neurite outgrowth inhibition protein A (Nogo‐A), and chondroitin sulfate proteoglycans,^[^ ^170^ ^]^ Brochier and colleagues reported that PARP1 was activated in primary cortical neurons exposed to these growth‐inhibiting agents (PAR primarily in neuronal cytoplasm in this study) and that PARP1 deletion or pharmacological inhibition promoted neurite outgrowth compared to respective control cultures.^[^ ^171^ ^]^ These data suggest that PARP1 or its PARylation activity may be an intrinsic molecular target for axonal regeneration.

The homeostasis of protein PARylation, which is cooperatively controlled by PARP1 and PARG activity (Figure 1), may be a promising target for axonal regeneration. Increasing PARylation by depleting the de‐PARylation enzyme PARG was reported to inhibit axonal regeneration of GABA neuron in axotomized C. elegans whereas decreasing PARylation by deleting C. elegans PARP1 or PARP2 promoted axonal regeneration of GABA neurons.^[^ ^172^ ^]^ These observations indicate that neuronal PARylation level controls GABAergic axonal regeneration in invertebrate CNS. Furthermore, shRNA‐mediated PARP1 knockdown also promotes axonal regeneration of primary cortical mouse neurons in an in vitro axonal injury/regeneration assay,^[^ ^172^ ^]^ suggesting that the regeneration‐promoting effects of inhibiting PARylation are conserved in mammalian neurons. However, subsequent experimental data from the same group demonstrated that inhibiting cellular PARylation by PARP1 deletion or pharmacological PARP1 inhibition fails to enhance axonal regeneration or functional recovery after adult mammalian CNS injury,^[^ ^173^ ^]^ indicating an intrinsic difference in the molecular requirement for axonal regeneration between invertebrate and vertebrate CNS and between in vitro and in vivo contexts. It is unclear whether Wang et al., used mice of male, female, or both for axonal injury/regeneration in the traumatic injury models.^[^ ^173^ ^]^ Taken together, future studies are needed to interrogate the role of PARP1 in axonal degeneration and regeneration in the context of CNS inflammatory demyelinating injury.

## 7 Role of PARP1 in OL Damage, Demyelination, and Remyelination: Puzzles Remain

OLs and myelin are the primary targets in MS. Remyelination failure has been proposed as one of the major reasons for MS disease progression.^[^ ^174^ ^]^ Consistently, promoting OL regeneration and remyelination has been shown to prevent axonal loss and improve functional recovery in EAE mouse model of MS.^[^ ^2a^ ^]^ PARP1's enzymatic activity has been reported to be dysregulated in oligodendroglial lineage cells in MS and animal models.^[^ ^15^, ^16^ ^]^ Hence, the future translation of PARP1 inhibition into MS therapies must consider the potential role of PARP1 in OL damage and regeneration. Unfortunately, the function of PARP1 in oligodendroglial biology and pathology remains incompletely defined and very limited amount of data have been thus far available in the field.

### 7.1 PARP1 in OL Differentiation and Developmental Myelination

Using PARP1‐KO mice, we made the first observation that PARP1 deficiency switched the fate of NSCs from neurogenesis to gliogenesis.^[^ ^48^ ^]^ Despite increased generation of OL progenitor cells (OPCs), oligodendroglial myelination was impaired in the brain of PARP1 KO mice, as evidenced by thinner subcortical white matter tract and decreased MBP immunostaining in both male and female PARP1 KO animals.^[^ ^48^ ^]^ The reduced myelination was proposed to represent a negative feedback to the enhanced generation of OPCs from PARP1‐deficiency NSCs.^[^ ^48^ ^]^ Alternatively, PARP1‐deficient OPCs may exhibit impaired capability to differentiate into OLs, or subsequent myelination in global PARP1 KO mice. Oligodendroglial lineage‐specific PARP1 KO paradigm will help determine these possibilities.

Recently, Baldassarro and colleagues reported that inhibiting PARP1 activity by PJ34, thieno[2,3‐c]isoquinolin‐5‐one (TIQ‐A), and Olaparib in micromolar doses caused cytotoxicity to OPCs in a mixed glial cell culture derived from fetal but not adult brain,^[^ ^102^ ^]^ suggesting that PARP1 activity is required for the normal survival and growth of OPCs in the dish. The differential toxicity of fetal versus adult OPCs to PARP1 inhibitors is likely due to the higher PARP1 expression and activity in fetal OPCs than adult OPCs as reported.^[^ ^102^ ^]^ Interestingly, when fetal OPCs were maintained in PARP1 inhibitor‐containing differentiation medium, significantly lower percentage of differentiated OLs positive for myelin protein (MBP and CNP) was observed in the inhibitor group than that in the vehicle group.^[^ ^102^ ^]^ Together, previous data from our own group and others indicate that PARP1 and its activity may play an essential role in differentiation of OPCs into OLs and/or subsequent myelination by myelinating OLs. Given the ubiquitous expression of PARP1 in all type of cells and multiple sequential steps of oligodendroglial lineage maturation and progression,^[^ ^117b^ ^]^ it is important and necessary to use oligodendroglial lineage‐specific and/or oligodendroglial stage‐dependent PARP1 KO systems to define the role of PARP1 in OL differentiation and myelination in the brain. Furthermore, it remains enigmatic whether PARP is physiologically activated in oligodendroglial lineage cells in vivo and whether PARP1 activity in OPCs and/or OLs, if any, play a role in their survival or death under physiological conditions.

### 7.2 PARP1 in OL Damage and Demyelination

Using postmortem brain of MS patients, previous study reported that PAR (the product of PARP1 enzymatic activity) was located primarily in OLs and as well as macrophages/microglia and astrocytes in MS demyelination plaques.^[^ ^15^ ^]^ The density of PAR^+^ cells was found in higher density in pattern III plaques (i.e., primary oligodendrogliopathy) than pattern II plaques and positively correlated with the density of cells positive for nuclear AIF, a unique hallmark of PARP1‐mediated cell death.^[^ ^15^ ^]^ This clinical observation provides the rationale for studying the role of PARP1 in demyelination. Using cuprizone‐induced primary demyelination model (see Section 1.1), Veto and colleagues reported that pharmacologically inhibiting PARP1 enzymatic activity by 4HQ protected mice against demyelination and decreased caspase‐independent AIF‐mediated cell death in the brain.^[^ ^15^ ^]^ The authors concluded that PARP1 activation‐induced OL death and aggravated experimental demyelination through AIF‐mediated parthanatos. This conclusion is consistent with the observations derived from in vitro neuron studies that PARP1 activation mediates AIF‐dependent cell death.^[^ ^84^ ^]^ However, several limitations exist compromising data interpretation for the proposed role of PARP1 activation in mediating oligodendroglial damage and demyelination.

First, it remains enigmatic whether the observed protective effect of PARP1 inhibition is directly derived from oligodendroglial PARP1 or indirectly from PARP1 in other cells. The authors assumed but did not prove that PARP1 was activated primarily in OLs during cuprizone‐induced demyelination. We found that, at the histological level, PAR was markedly upregulated in the corpus callosum during demyelination at 3 weeks of cuprizone diet, which is consistent with the Western blot evidence presented by Veto and colleagues.^[^ ^15^ ^]^ In stark contrast, PAR^+^ cells were rarely co‐labeled with the pan‐oligodendroglial lineage marker SOX10, instead, appeared correlated with dense CD68^+^ activated microglia (**Figure** **4**A). These observations indicate that the reported oligodendroglial protective effect^[^ ^15^ ^]^ may indirectly result from PARP1 inhibition in microglia. Previous studies have reached a consensus that PARP1 promotes microglial proliferation and activation in response to various stimuli.^[^ ^168^, ^169^, ^175^ ^]^ At the functional level, microglial activation is not only necessary but also sufficient for OL death and demyelination in cuprizone demyelinating model.^[^ ^24^ ^]^ It is tempting to hypothesize that PARP1 chemical inhibitors, applied throughout the demyelination stage,^[^ ^15^ ^]^ interfere with microglial activation, which indirectly protects OLs and myelin against cuprizone‐induced damage. It would be very important to define the role of microglial‐specific PARP1 in microglial activation and oligodendroglial demyelination in cuprizone demyelination model, or in EAE autoimmune model. In this regard, it remains unclear whether PARP1 activation in oligodendroglial lineage cells, if any, plays a role in OL death and demyelination.

Second, AIF nuclear translocation is a hallmark of PARP1‐mediated cell death. No clear evidence of AIF nuclear translocation was presented to indicate PARP1‐mediated OL death.^[^ ^15^ ^]^ At the histological level, the mitochondrial AIF protein was observed rarely in the nucleus where PAR was highly concentrated (Figure 4B), suggesting that PARP1 activation may play a death‐independent role during demyelination/remyelination in the cuprizone model. Recent data suggest that cuprizone‐induced OL damage and demyelination are mediated by ferroptosis,^[^ ^176^ ^]^ an ROS‐dependent form of cell death which is characterized by iron accumulation and lipid peroxidation and mechanistically distinct from PARP1‐mediated parthanatos.^[^ ^177^ ^]^ Although peroxynitrite and nitrosative stress play an important role in OL death in MS and cuprizone models, PARP1 activation is reported to be dispensable for peroxynitrite‐induced OL toxicity in primary OL culture.^[^ ^91^ ^]^ Scott et al., reported that peroxynitrite treatment (60–500 µM) induced a concentration‐dependent reduction in the mitochondria function, DNA damage, PARP1 activation, and cell death of OLs. However, PARP1 inhibition by small compounds and PARP1 deletion did not reduce OL death in response to different concentration of peroxynitrite.^[^ ^91^ ^]^ These observations suggest that PARP1 activation is dispensable for nitrosative stress‐induced cytotoxicity of OLs. Taken together, the role of PARP1 in oligodendrogliopathy is still controversial. More studies, particularly those employing in vivo genetic approaches, are necessary to define the role of PARP1 activation in OL death and demyelination in autoimmunity and toxin‐induced demyelination animal models.

As delineated in Figure 3, PARP1 is a co‐activator of NF‐*κ*B signaling pathway acting at the multiple levels of the signaling axis. It is conceivable that pharmacological PARP1 inhibition would dampen the transcriptional activity of NF‐*κ*B in OLs in response to demyelination insults. Previous genetic data from Lin and colleagues demonstrated that NF‐*κ*B activation is cytoprotective for OLs, as transgenic mice of oligodendroglial‐specific NF‐*κ*B inactivation exhibit greater degree of OL death in response to ectopic IFN‐*γ* expression in cuprizone model and are more susceptible to EAE than NF‐*κ*B intact mice.^[^ ^93^, ^178^ ^]^ Furthermore, NF‐*κ*B activation mediates the cytoprotective effects of pancreatic ER kinase (PERK) on OLs in EAE.^[^ ^179^ ^]^ In this regard, we instead hypothesize that PARP1 activation may be cytoprotective for OLs and that oligodendroglial‐specific PARP1 inhibition renders OLs more susceptible to inflammatory demyelination in MS and EAE where NF‐*κ*B plays an important role in the disease pathogenesis.

### 7.3 PARP1 in OL Regeneration and Remyelination

While the role of PARP1 in OL death under various injury conditions was a focus of previous studies^[^ ^180^ ^]^ (see Section 7.2), its role in OL regeneration and remyelination receives no attention and few experimental data are available in the field. A PubMed search using the key works of PARP1 (or PARylation), OLs, and remyelination retrieved a few relevant publications. One publication reported that PARP1 expression was markedly upregulated in brain white matter OLs of major depressive disorder (MDD) patients compared to controls and proposed as a protective mechanism in response to oxidative stress‐induced DNA damage which was observed in MDD brain.^[^ ^181^ ^]^ In MS patients, PARP1 expression appears downregulated in chronic inactive plaques compared to controls as evaluated by proteomic profiling.^[^ ^182^ ^]^ Although the cell type(s) with PARP1 downregulation has yet to be determined, this observation indicates that augmenting PARP1 expression or enhancing PARP1‐mediated PARylation may be a hypothetical avenue to decrease demyelination and/or promote remyelination. Interestingly, non‐colocalization of PAR and AIF in DAPI^+^ nuclei (Figure 4B) suggests that PARP1 activation may be required for OL regeneration and remyelination. During the preparation of this manuscript, a recent work by Wang and colleagues^[^ ^128^ ^]^ reported that pharmacologically inhibiting PARP1 activity by 4HQ inhibited OL regeneration and remyelination in cuprizone mouse model, suggesting that PARP1 activation is necessary for remyelination. It remains unknown whether the observed effect^[^ ^128^ ^]^ is derived from PARP1 inhibition in oligodendroglial lineage cells or indirectly from PARP1 inhibition in other lineage cells. Defining the temporal dynamics and cell types of PARP1 activation during demyelination and remyelination stages will provide novel insights into this question. To prove a direct role of PARP1 in remyelination, oligodendroglial‐specific conditional knockout systems are needed given the ubiquitous expression of PARP1 in all mammalian cells.

## 8 Role of PARG in Neurodegeneration, Neuroinflammation, and Oligodendroglial Myelination: Implications in MS and EAE

PARG is the primary catabolic enzyme which cleaves PAR polymers. Under homeostatic conditions, PARG and PARP1 work cooperatively to control cellular PARylation level (Figure 1). A growing number of studies using pharmacological PARG inhibition have suggested that PARG exhibits neurotoxic effect on ischemic brain injury. Inhibition of PARG activity by gallotannin reduced cell death of primary neurons and astrocytes elicited by oxidative stress (H_2_O_2_), excitotoxicity (NMDA), or genotoxic stress (MNNG).^[^ ^183^ ^]^ Pre‐ or post‐ischemia treatment with *N*‐bis‐(3‐phenyl‐propyl)9‐oxo‐fluorene‐2,7‐diamide (GPI 16552), a PARG inhibitor, was shown to diminish brain infarct volume in a rat model of focal brain ischemia.^[^ ^184^ ^]^ Furthermore, Wei and colleagues reported that intranasal administration of gallotannin after focal brain ischemic injury abolished AIF nuclear translocation, decreased ischemic brain damage, and promoted neurological recovery in a mouse model of focal brain ischemia.^[^ ^185^ ^]^ The exact molecular mechanisms underlying the reported protection remain enigmatic yet appear multi‐modal; preservation of cellular NAD^+^ pool was proposed as the major one. PARG inhibition was shown to decrease cellular PAR turnover, which leads to increased auto‐PARylated PARP1 (PARP1 is the major PARylation acceptor protein). Increased PARP1 auto‐PARylation provides a negative feedback to limit PARP1 enzymatic activity which in turn reduces NAD^+^ consumption and promotes cell survival. Unfortunately, no direct data are available to support the proposed mechanism.

Different from pharmacological PARG inhibition, studies using genetic PARG depletion, however, suggest a neuroprotective role of in ischemic brain injury. The murine *Parg* gene, consisting of 18 exons, produces two PARG isoforms via alternative splicing and translation initiation: the canonical 110 kD isoform (PARG_110_) which is localized primarily in the nucleus and accounts for most PARG activity and the 60 kD isoform (PARG_60_) in the nucleus and cytoplasm. Deletion of exons 2 and 3 at the genomic level leads to loss of the 110 kD isoform while exons 2–4 deletion leads to loss of both isoforms. Mice with germline deletion of both isoforms are early embryonic lethal due to massive PAR accumulation and cell apoptosis^[^ ^186^ ^]^ while those of PARG_110‐_specific deficiency are viable and fertile.^[^ ^187^ ^]^ PARG_110_‐deficient mice are hypersensitive to DNA alkylating agents and susceptible to LPS‐induced endotoxic shock^[^ ^187^ ^]^ indicating that PARG_110_ is cytoprotective in response to various stress conditions. Furthermore, when subjected to focal ischemia brain injury, PARG_110_‐deficient mice exhibited higher PAR level in the brain (which is expected, due to impaired de‐PARylation activity) and developed larger cerebral infarct volume than PARG_110_‐intact mice.^[^ ^188^ ^]^ Interestingly, the expression or activation of caspase 3, PARP1, Akt, and IL1*β* or iNOS was comparable between PARG_110_‐deficient and sufficient mice.^[^ ^188^ ^]^ These data indicate that the observed neuroprotective role of PARG in ischemia brain injury is unlikely due to alterations of caspase 3‐mediated apoptosis, PARP1‐mediated parthanatos, Akt‐mediated survival pathway, or IL1*β*‐mediated inflammatory response. Furthermore, the level of NAD^+^ and ATP in ex vivo brain slices was not affected by PARG_110_ deficiency under both basal conditions and MNNG‐stimulated conditions,^[^ ^188^ ^]^ suggesting that the increased ischemia brain injury inPARG_110_ deficient mice is independent of NAD^+^ depletion and energy deficit. The reasons for the conflicting outcomes between PARG chemical inhibition and congenital PARG_110_ depletion may be multi‐factorial. For example, PARG inhibitors may block the activity of both PARG_110_ and PARG_60_ isoforms, thus having a prominent impact on PAR catabolism and cellular NAD^+^ level compared to PARG_110_ depletion. Consistently, PARG_110_ depletion did not alter NAD^+^ levels in the brain slices upon exposure to MNNG^[^ ^188^ ^]^ whereas PARG chemical inhibition rescued cellular NAD^+^ level upon exposure to MNNG.^[^ ^183a^ ^]^ The differential influence on NAD^+^ levels may account for the cytoprotective effect of chemical PARG inhibition. In addition, the specificity and efficacy of PARG inhibitors may be a concern,^[^ ^189^ ^]^ raising the cautions when interpreting experimental data obtained from pharmacological PARG inhibition both in vitro and in vivo.

PARG inhibition may exert therapeutic potential in immunomodulation. In a mouse model of traumatic spinal cord injury (SCI), PARG inhibition by GPI 16552 or PARG_110_ deficiency decreased the severity of SCI, diminished neutrophil infiltration and TNF*α* and IL1*β* expression, and attenuated apoptotic cell death as well.^[^ ^190^ ^]^ These observations suggest that different from ischemic brain injury, PARG activity aggravates tissue damage presumably through mediating immune infiltration and inflammatory cytokine expression under traumatic CNS injury conditions.

The role of PARG in oligodendroglial myelination remains incompletely defined. We recently reported that cellular PARylation plays an important role in the differentiation of OPCs into OLs.^[^ ^128^ ^]^ We showed PARG inhibition and silencing increased oligodendroglial PARylation level and promotes OPC differentiation. This finding suggests that inhibiting PARG or enhancing PARylation may promote OPC differentiation into OLs, a process which is usually blocked, leading to remyelination failure, in MS and EAE animal model.

Taken together, whereas the role of PARG in ischemic brain injury has been intensively studies, its therapeutic potential in MS pathogenesis and disease regulation remains to be defined. It is yet to be determined whether PARG inhibition yields opposite outcomes to PARP1 inhibition in modulating neuroinflammation, neurodegeneration, and demyelination given the reverse effect of PARG and PARP1 on PARylation. Since cellular PARylation is a coordinated process catalyzed by PARP1 and PARG, it is possible that PARG may be activated in the cells with simultaneous PARP1 activation, which has been identified in multiple cell types of immune and neural cells. Given the early lethality of global PARG KO mutants and off‐target effect of small inhibitors, it is important to employ PARG conditional KO and interrogate the role of PARG in neuroinflammation, neurodegeneration, and demyelination in MS and EAE models.

## 9 Conclusions, Perspectives, and Future Directions

1) Consensus has been reached that PARP1 plays an important role in maintaining genomic stability, regulating gene expression, and modulating cellular NAD^+^/ATP homeostasis.

During the past two decades, a growing number of preclinical animal studies have demonstrated that PARP1 inhibition may exhibit therapeutic potential in reducing disease severity of ischemia brain injury, neurodegenerative disorders, and possibly MS. PARP1 inhibition was reported to suppress immune activation and neuroinflammation. However, preclinical MS animal studies have reported discrepant or even contradictory observations. The conflicting reports of EAE disease severity between PARP1 chemical inhibition and PARP1 genetic deficiency make it necessary to revisit the pathophysiological role of PARP1 in peripheral immune activation and neuroinflammation in the context of MS animal models. One of the prevailing rationales underlying the protective effect of PARP1 inhibition on EAE is through inactivating NF‐*κ*B, a master regulator of immune response and neuroinflammation (Figure 3). However, previous data indicate NF‐*κ*B inactivation could attenuate or aggravate EAE disease severity depending on different cell types (see review^[^ ^93^ ^]^). We hypothesize that PARP1 may exert a cell‐dependent role in pathogenesis and disease progression of MS and animal models. In this regard, cell‐specific and time‐conditional PARP1 genetic models are necessary to define the therapeutic potential of PARP1 inhibition in MS and animal models.

2) Excessive PARP1 hyperactivation results in acute necrotic cell death, a process referred to as caspase‐independent and AIF‐mediated parthanatos, presumably by rapid NAD^+^ depletion and energy failure. This concept provides a rationale for testing the therapeutic potential for PARP1 inhibition in cytoprotection (neurons and OLs) both in vitro and in vivo in previous studies. We propose that the level of PARP1 activation in response to different injury severity determines cell fate of death and survival. Experimental data have demonstrated that PARP1 activity exhibits neuroprotection against mild‐to‐moderate oxidative stress, which likely mimics the progressive oxidative situation happened in MS patients. Our recent data demonstrated that PARP1 activity is dispensable for OL death, instead, it promotes OPC differentiation and remyelination in demyelination animal models. Furthermore, the essential role of PARP1 in DNA repair and cellular homeostasis must be considered. DNA damage, a robust trigger for PARP1 activation, is markedly increased in the CNS of EAE mice as assessed by DNA damage marker *γ*H2AX.^[^ ^53a^ ^]^ It is possible that PARP1 inhibition may disrupt PARP1‐mediated DNA repair and genomic integrity, leading to neuronal/oligodendroglial death and EAE disease aggravation. Experimental data are needed to define the biological effects of PARP1 deletion on neuronal and oligodendroglial death in EAE animal models. Moreover, potential sexual dimorphism of PARP1 inhibition in MS disease regulation and neuronal/glial cell death is still an open question and cannot be neglected prior to clinical translation.
3) Like PARP inhibition, PARG inhibition was also reported to exhibit therapeutic potential in cancer therapy and ischemic brain injury. The role of PARG and PARG‐mediated PARylation homeostasis in immune response, neuronal/axonal damage, and oligodendroglial damage and demyelination/remyelination remains enigmatic in the settings of MS and animal models. The reported neuroprotection of both PARP1 inhibition and PARG inhibition suggests that the cellular PAR turnover rate may determine the cell fate choices (death versus survival) in different neurological conditions including MS.
4) Therapeutic potential of PARP1 (or PARG) in MS may also depend on different stages of the disease. While most previous studies focused on the effects of PARP1 inhibition on acute neuroinflammation, its biological outcome on disease progression during the chronic neurodegenerative phase remains to be defined. It is generally accepted that MS disease progression is associated with remyelination failure and chronic neuronal/axonal degeneration. It would be important to study the role of PARP1 or PARP1/PARG‐controlled cellular PARylation in oligodendrogliopathy and neuropathy during the chronic phase of MS. It remains unknown whether PARP1 (or PARG) activation is beneficial or detrimental to OLs and neurons in the context of MS. Given all these questions discussed above, it is too soon to translate PARP1 inhibition into MS therapeutic strategies. The availability of *Parp1*‐floxed and *Parg*‐floxed transgenic animals will help define potential cell type‐dependent role of PARP1 (or PARG) in MS pathogenesis and disease modulation.

## Conflict of Interest

The authors declare no conflict of interest.
